# Artificial Intelligence for Computer-Aided Detection in Biomedical Applications

**DOI:** 10.3390/bioengineering13040448

**Published:** 2026-04-11

**Authors:** Lawrence Wing Chi Chan

**Affiliations:** Department of Health Technology and Informatics, Hong Kong Polytechnic University, Hong Kong SAR, China; wing.chi.chan@polyu.edu.hk

## 1. AI Bridging Innovation, Clinical Translation, and Future Horizons

Artificial intelligence (AI) plays an important role in bioengineering that has promoted a paradigm shift in how disease diagnosis, treatment planning, and patient monitoring are performed. From medical image enhancement to therapeutic response prediction, AI-driven tools are widely exhibiting their potential to streamline clinical workflows and improve patient outcomes. This Special Issue brings together impactful studies that not only showcase the remarkable advancements in the field but also critically address the persistent gaps that suppress widespread clinical adoption. These works signify a pivotal transition from algorithm development toward robust, explainable, and clinically aligned solutions.

## 2. Recent Advance and Persistent Gaps

We have witnessed the unprecedent breakthrough of deep learning in medical image analysis, fueled by the advanced model architectures such as U-Net, Vision Transformers, and the Segment Anything Model (SAM). Studies in this issue, such as the work by Kim and Song [1] on AI-augmented fundus disease screening and the comprehensive review by Mashekova et al. [2] on breast cancer detection across mammography, ultrasound, and thermography, exemplify the growing sophistication of AI in various modalities. Similarly, Wang et al. [3] demonstrated the utility of deep learning for grading cervical spinal stenosis, while Kang et al. [4] introduced a novel dual-channel network for Parkinson’s disease classification using transcranial sonography, and highlighted the expanding diagnostic reach of AI.

Despite these advances, there are still several crucial gaps. First, the generalizability of AI models across diverse populations, imaging protocols, and clinical settings remains a concern, which stems from small and homogeneous training datasets. Second, the “black-box” nature of many deep learning models impedes clinician trust and limits adoption. Third, the integration of multi-modal data, ranging from imaging to spatial omics, remains underexplored in unlocking holistic disease insights. Fourth, there is a notable delink between model performance on curated datasets and real-world clinical utility, substantiating the issues of image quality, workflow integration, and interpretability.

## 3. Addressing the Gaps: Contributions in This Special Issue

The articles assembled here directly tackle these challenges from multiple angles, providing a blueprint for the next generation of clinically viable AI systems.

1. Enhancing Data Quality and Model Robustness: A foundational challenge in medical AI is the quality of input data. Kong et al. [5] address this head-on by proposing a novel image-enhancement scheme that blends anisotropic diffusion, histogram equalization, and window width and level adjustment to improve the performance of the Segment Anything Model (SAM) for liver segmentation. Their work demonstrates that thoughtful preprocessing can significantly elevate segmentation accuracy, highlighting that data-centric approaches are as crucial as model innovation. Complementing this, Rodriguez Alonso et al. [6] tackle data scarcity in melanoma histopathology through synthetic data augmentation, showing how generative techniques can bolster model performance and reduce reliance on large, annotated datasets.

2. Bridging the Explainability Gap: For AI to earn clinicians’ trust, it must be interpretable. Cai et al. [7] provide a timely review of explainable AI (XAI) based on visual explanations in digestive endoscopy, mapping how techniques like saliency maps and attention mechanisms can demystify model decisions. This focus on transparency is echoed in the work by Wang [8], who discusses integrating AI with precision therapeutics for age-related macular degeneration, emphasizing that explainable models are essential for aligning AI predictions with clinical reasoning.

3. Advancing Multi-Modal and Longitudinal Models: Moving beyond single-modality analysis, Kgabeng et al. [9] explore the integration of spatial omics with deep learning to predict cardiomyocyte differentiation efficiency, demonstrating a pioneering step toward predictive models that leverage molecular and spatial data simultaneously. In a different angle, Willms et al. [10] develop a longitudinal dynamic risk score for delayed cerebral ischemia after subarachnoid hemorrhage, showcasing the value of time-series data in capturing evolving clinical states. Similarly, Liu et al. [11] present an adaptive gait event identifier for real-time analysis, emphasizing the importance of dynamic, temporal modeling in functional assessments.

4. Improving Clinical Alignment and Workflow Integration: Several studies focus on embedding AI into concrete clinical tasks. Xie et al. [12] introduce a novel deep learning system and the TB-Seg dataset for evaluating bone tunnels in anterior cruciate ligament reconstruction, addressing a specific surgical outcome. Tandon et al. [13] develop a deep learning classifier using chest radiographs to predict extubation success in mechanically ventilated patients—an application that directly impacts critical care decision-making. Park et al. [14] contribute a weakly supervised method for quantifying interval changes in interstitial lung disease on chest radiographs, aligning AI output with the radiologist’s task of assessing disease progression over time.

5. Refining Evaluation and Addressing Underrepresented Tasks: Park’s comprehensive review [15] of performance metrics for computer-aided detection systems provides a critical framework for standardizing evaluation, ensuring that models are assessed on clinically meaningful outcomes. Meanwhile, Lee et al. [16] tackle a niche but important problem: exhale-focused thermal image segmentation using optical flow and Transformer networks, demonstrating the value of tailoring AI to specific physiological signals.

## 4. Future Research Directions

Looking forward, the convergence of insights from this Special Issue points to several promising avenues:

1. Foundation Models and Adaptation: The success of models like SAM in general segmentation, as explored by Kong et al. [5], suggests that foundation models fine-tuned for medical domains (e.g., MedSAM) will become increasingly prevalent. Future work should focus on efficient adaptation strategies and domain-specific enhancements to bridge the gap between general-purpose models and clinical nuances.

2. Multi-Modal and Omics Integration: The integration of imaging with genomics, spatial transcriptomics (as in Kgabeng et al. [9]), and clinical data holds the key to precision medicine. Developing architectures that seamlessly fuse these disparate data types will enable more holistic predictive models for disease progression, treatment response (e.g., Huang et al. [17] on PD-L1 inhibitor responses), and patient stratification.

3. Explainability and Human–AI Collaboration: Future systems must move beyond post hoc explanations to inherently interpretable models. Research should prioritize user-centered design, evaluating how AI predictions and explanations influence clinician decision-making in real-world settings.

4. Robustness and Generalization: Addressing domain shifts, dataset biases, and variability in image acquisition (as highlighted in Mashekova et al. [2] and Zhang et al. [18] on ultrasound segmentation) requires the development of robust training strategies, including domain adaptation, synthetic data generation, and federated learning across institutions.

5. From Prediction to Intervention: The ultimate goal is to close the loop from diagnosis to treatment. Future work should focus on embedding AI within therapeutic workflows in surgical guidance, adaptive radiotherapy, or closed-loop monitoring systems to directly impact patient outcomes.

In conclusion, this Special Issue captures a vibrant moment in the field of AI in bioengineering. By addressing foundational challenges in data quality, model interpretability, multi-modal integration, and clinical alignment, these studies collectively pave the way for AI systems that are not only powerful but also trustworthy and actionable. We hope this collection inspires continued innovation and fosters the interdisciplinary collaborations essential for translating AI promise into tangible patient benefit.

## Data Availability

No data is presented in this article.
